# The role of duty, gender and intergenerational care in grandmothers’ parenting of grandchildren: a phenomenological qualitative study

**DOI:** 10.1186/s12912-024-02151-0

**Published:** 2024-07-15

**Authors:** José Miguel Mansilla-Domínguez, Ana María Recio-Vivas, Laura Lorenzo-Allegue, José Miguel Cachón-Pérez, Laura Esteban-Gonzalo, Domingo Palacios-Ceña

**Affiliations:** 1 Department of Nursing, Faculty of Biomedical and Health Sciences, European University of Madrid, Madrid, Spain; 2 Departament of Nursing, Universidad Complutense de Madrid, Madrid, Spain; 3 Research Group of Humanities and Qualitative Research in Health Science, Department of Physical Therapy, Occupational Therapy, Physical Medicine and Rehabilitation, Universidad Rey Juan Carlos (Hum&QRinHS), Alcorcón, Spain

**Keywords:** Child care, Grandparents, Gender role, Qualitative research

## Abstract

**Objectives:**

The aim of this study was to describe grandmothers’ experiences of taking care of their grandchildren in terms of their care-giving tasks, motivations and emotions.

**Methods:**

A qualitative phenomenological study was conducted. Purposive sampling was used, based on the relevance of the research question. Seventeen participants were included, women ≥ 65 years old, grandmothers who care for their grandchildren at least 10 h per week and who attended the Nursing units of the Primary Care Health Centers (Madrid Public Health Service). Seventeen in-depth interviews were conducted. The interviews were audio-recorded, transcribed verbatim and thematic analysis was carried out from the perspective of hermeneutic phenomenology. For the analysis, the Excel program was used to organize and share the coding process. Also, we followed COREQ guidelines.

**Results:**

Four main themes were identified: (a) Care out of obligation, where participants feel an obligation to help their children by caring for grandchildren, regardless of their number, and prefer to do so voluntarily; (b) Care out of responsibility, where grandmothers see their role as a responsibility that includes saving costs by caring for grandchildren and facilitating their children’s work life balance; (c) Care as a social duty, reflecting a moral commitment inherited from their mothers to help future generations; and (d) Construction of care from a gender perspective, where grandmothers, as women, primarily assume the care and upbringing of grandchildren.

**Discussion:**

Our results contribute to increase knowledge about childcare provided by grandmothers to their grandchildren. Grandmothers become fundamental pillars of families by helping their children balance family and work. Behind this care there is a strong sense of obligation, duty and generational responsibility. Grandmothers’ help presents differences in the distribution of tasks and care by sex. Identifying factors that motivate grandmothers to care for their grandchildren helps nurses to perform higher quality comprehensive care.

**Supplementary Information:**

The online version contains supplementary material available at 10.1186/s12912-024-02151-0.

## Introduction

Aging populations as well as social and demographic change have led to the modification and reorganization of family relationships and particularly those taking place between grandparents and their grandchildren [1]. Previous studies [2–4] show increased involvement of grandmothers in the care of grandchildren, a shared experience among the elderly. [2–4].

Grandparents have always played a fundamental role in the transmission of values to their grandchildren. But this role has changed as they have taken over the care, upbringing and education of their grandchildren [5]. Grandmothers/grandfathers have become family welfare providers in a context of economic and social crisis [6]. In recent decades, the percentage of grandparents aged 65 or older who report caring for their grandchildren has doubled [7]. In their work on grandparents’, [8] Gleeson et al. [8] show how commitment to caring for grandchildren has increased from 11% in 2010 to 17% in 2018. Similarly, The Survey of Health, Ageing and Retirement in Europe [9] describes how 22.07% of grandparents care for their grandchildren. This same report found Spain to be the country where there are fewer grandparent caregivers, but those who do provide care, do so with greater intensity than grandparents in other European countries. Spanish grandmothers devote an average of 7.1 h a day to caregiving (including weekends), compared to 4.9 h a day in Europe. Moreover, this care is provided on a voluntary basis [10].

Voluntariness is sometimes experienced by grandmothers as an obligation, influenced by the values and education they have received and acquired during their lives [11]. In addition, there are gender differences in the distribution of caregiving tasks and responsibilities among grandmothers and grandfathers [12]. Grandmothers assume the main responsibility for caregiving and adopt the role of the caregiver, while grandfathers take on a secondary or complementary caregiver role [12]. Thus, unequal distribution of caregiving responsibilities during their own parenthood is perpetuated in the care of grandchildren [13].

Previous qualitative studies [7] have described how grandmothers who cared for their grandchildren were actively involved in their socialization and education. Lee & Blitz [14] note how grandmothers take on a greater burden because of their greater involvement in caring for grandchildren. Despite this burden, the reasons that influence them to accept the care of their grandchildren are based on the voluntariness of the care, and the acquired gender role [15]. Sometimes this decision is made jointly with the children’s parents or in response to a sense of obligation [16].

In contrast, the evidence regarding grandmother caregiving is scarce. Therefore, what are the lived experiences of grandmothers with grandchildren’s care? What are the reasons for this care? The objective of the present study was to describe the lived experiences of grandmothers who take care of their grandchildren regarding their care, motivations, and feelings.

## Materials & Methods

### Design

A qualitative phenomenological study was conducted based on Husserl’s framework [17–20]. In the field of qualitative studies, phenomenology attempts to understand other people’s lived experiences by using first-person narratives and other sources such as personal letters, diaries and drawings [21–23]. Thus, in phenomenology, two approaches in particular are influential in healthcare: (a) descriptive phenomenology has the closest connection with Husserl’s original conception of phenomenology and focuses on creating detailed descriptions of the specific experiences of others [24–26] (ref) and (b) interpretative or hermeneutic phenomenology, which seeks to understand the nature of human beings and the meanings they bestow upon the world by examining language in its cultural context [22]. Husserl indicates the need to “retain” beliefs (bracketing) which, using the phenomenological reduction approach, would allow a critical examination of the phenomena without the influence of the researcher’s own beliefs [23]. In our study, bracketing was achieved by carefully recording the positions taken by the researchers beforehand and by using in-depth interviews as the main data collection tool [19, 21]. In this way, we sought to avoid the researcher’s influence on the data and reveal the nature of the phenomenon through the patients’ accounts [23].

This study was carried out according to the Consolidated Criteria for Reporting Qualitative Research [27]. The study protocol was approved by the Ethical Committee of the Universidad Rey Juan Carlos (code: 01/2015) and the Local Ethical Committee of South Research-Madrid Health Service (code: 08/2012).

### Research team, reflexivity and context

All participants provided oral informed consent prior to their inclusion. All eight participating researchers (five women) were research nurses and university professors, experienced in health science investigation. The study protocol was audited by an external researcher.

Prior to the study, the positioning of the researchers (Table 1) was established through two briefing sessions addressing the theoretical framework, the context, their prior experience and their motivation for the research [21]. Los investigadores no tenían conflicto de intereses con las participantes.

**Table 1:** Research team, context and reflexivity

Theoretical framework	The theoretical framework was interpretivist. From this perspective, human actions are meaningful, and the goal of interpretive inquiry is the understanding of how people interpret the meaning of this social phenomena.
Beliefs and positioning	Intergenerational care in grandmothers’ parenting of grandchildren can be an element that gives meaning to your life, with which you feel competent, useful and integrated in society. The acquisition of the role of caregiver can become part of their personal identity, contributing benefits to their mental health and having a positive impact on other areas of grandmothers´ life.
Context	In Spain, grandmothers can assume the care of grandchildren when, due to different circumstances (work, separation, etc.), the parents are unable to care for their children. This means that grandmothers are sometimes considered an informal care resource; one in four grandmothers who care for their grandchildren invests many hours of her life in caring for the grandchildren, making it difficult or forcing her to abandon other personal activities in her life.
Motivation for the research	To explore intergenerational care in grandmothers’ parenting of grandchildren through participants’ first-hand experience. To describe and understand their experience to those matters they consider relevant to their lives and, thus, improve healthcare.

### Participants and sampling strategies

Purposive sampling was used, based on relevance to the research question (not clinical representativeness [28]. In phenomenology, it is necessary to include those people who experienced the phenomenon under study [20, 29]. In this case, participants who had relevant information about the phenomenon (caring for grandchildren) were included.

Sampling and data collection was pursued until the researchers achieved information redundancy, at which point no new information emerged from the data analysis [28], and/or when the information collected did not contribute anything new to the development of the properties and dimensions of the analysis [28]. In our study, this situation occurred after including 17 participants.

The study subjects included females ≥ 65 years old, grandmothers who care for their grandchildren at least 10 h per week, are retired from formal employment and do not live in the same household as their grandchildren. Exclusion criteria were grandmothers who cared for their grandchildren less than two days a week and/or less than one hour a day. The inclusion criterion determined by hours is based on the report “Productive ageing: Grandparents’ provision of care to grandchildren. Implications for their health and well-being” for the Institute of Elderly and Social Services (IMSERSO) of the Spanish Ministry of Labor and Social Affairs of 2008 [30], where it reflects that grandmothers who provide prolonged and intense care with great dedication to their grandchildren exceed at least 10 h per week.

The recruitment process of the participants consisted of an initial phase, where the Heads of Nursing and Centre Directors of the Health Centers of Pinto (Madrid) were contacted and a presentation of the project was made. The Heads of Nursing provided the telephone numbers of the grandparents recruited by Nursing and Medical professionals from the health centers in order to subsequently establish contact with the participants, thus guaranteeing a less invasive entry into the study by the researcher. The interviews were carried out in the Aula of the health center for two months, with all participants attending alone.

### Data collection

Based on the phenomenological design, first-person data collection tools (in-depth interviews) and researchers field notes were used simultaneously [20]. Unstructured in-depth interviews were used as the main tool for data collection. The interview started with an open question: ‘What is your experience with the care of your grandchildren? Thereafter, the researchers listened carefully, noted key words and topics identified in the subjects’ responses and used their answers to ask for and clarify content [20]. The interviews followed a guide created exclusively for this study (supplementary material- Appendix 1). In this way, relevant information was collected from the perspective of the participants. Researchers also used prompts or probes during the interviews: (a) to encourage the participants to provide more detail (‘Can you tell me a bit more about that?’), (b) to encourage the participant to keep talking (‘Have you experienced the same thing since?’), (c) to resolve confusion (paraphrasing of something that the patient had said) and (d) to show full attention by the researcher (‘That’s really interesting, please tell me more’) [20].

All interviews were tape-recorded and transcribed verbatim. Each of them ranged from 45 to 75 min (mean 57.85; SD 5.60). No third party was present in the interviews.

### Analysis

**Fig. 1:**
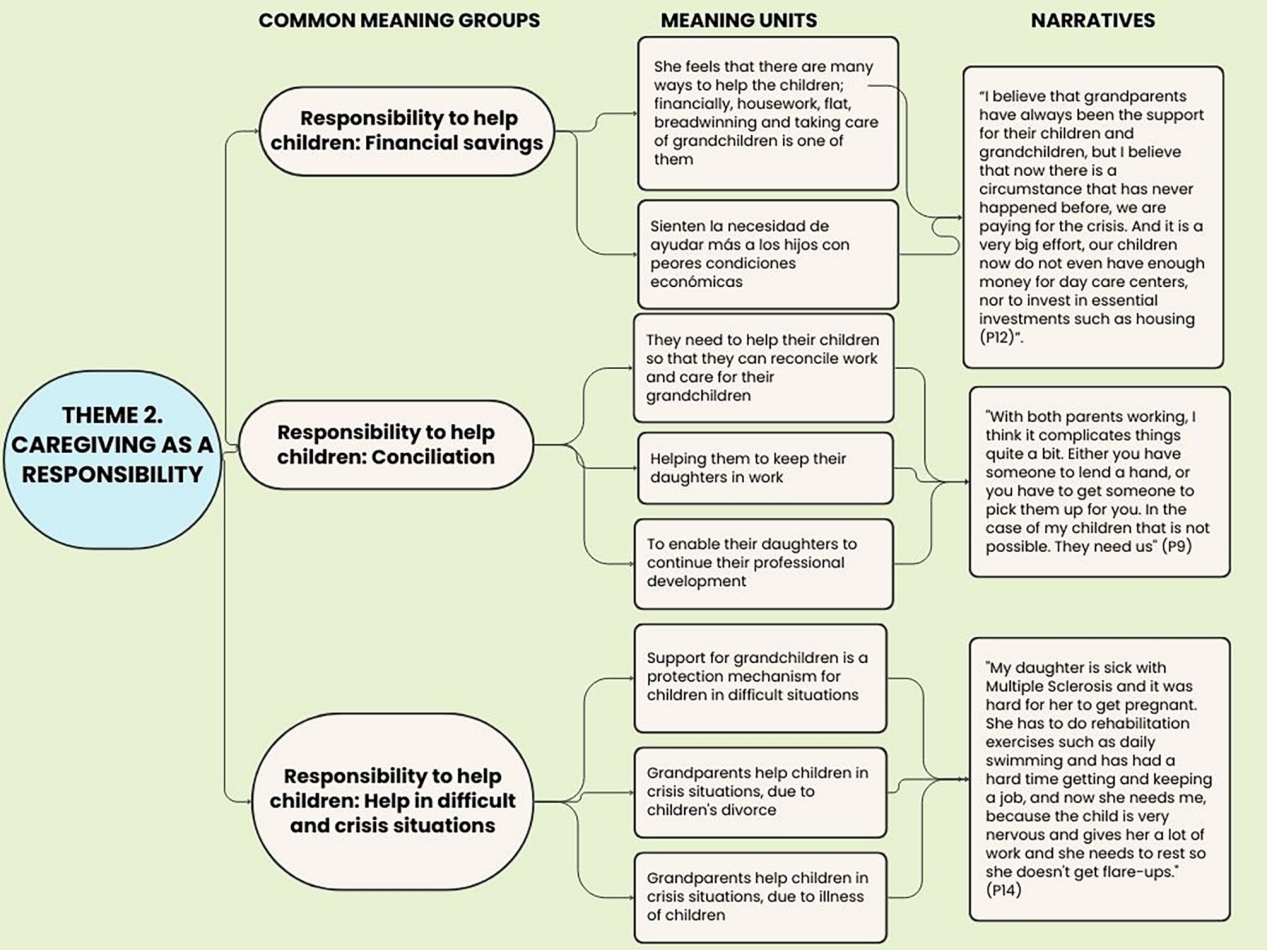
Example of analysis and coding procedure

First, a complete and literal transcription of each interview and the researchers’ field notes was drafted. Subsequently, an inductive analysis of the data was conducted [20] by two researchers (JMMD, DPC). During the data analysis stage, a model proposed by Amedeo Giorgi was used [31] which distinguishes 5 stages of data processing: (1) data collection; (2) reading literal transcription of the interviews; (3) breaking down the descriptions into separate units in order to identify the relevant meaning units for the phenomenon under study; (4) data organization and listing from the perspective of the discipline using an encoding process and, finally, (5) data synthesis and summarisation in order to communicate these to the scientific community. The breaking down and encoding process consisted of identifying the most descriptive content in order to obtain meaningful units and then reducing and identifying the most common meaningful groups. In this manner, groups of meaning units were formed, i.e., similar points or content allowing for emergence of themes that described the study participants’ experience Fig. 1.

For clarity, a matrix was built with results obtained from the analysis. Joint team meetings were held to display, combine, integrate, and identify themes. In case of difference of opinions, theme identification was based on consensus among research team members. No qualitative software was used to analyse the data. The Excel program (Microsoft Corp, Redmond, WA) was used to organize and share the coding process. In addition, researchers used the same Excel template shared through OneDrive (Microsoft Corp, Redmond, WA) to modify, highlight, and extract the results of the coding process simultaneously as part of a network.

### Rigor

The Lincoln and Guba criteria were used (Table 2) to establish data trustworthiness [32].

**Table 2:** Trustworthiness criteria

Criteria	Techniques performed
Credibility	Investigator triangulation: Team meetings were performed in which the analyses were compared, and categories were identified. Triangulation of methods of data collection: in-depth interviews and researcher field notes were retained. Member checking: this consisted of asking the participants to confirm the data obtained at the stages of data collection and analysis.
Transferability	In-depth descriptions of the study were performed, providing details of the characteristics of researchers, participants, contexts, sampling strategies, and the data collection and analysis procedures.
Dependability	Audit by an external researcher: an external researcher assessed the study research protocol, focusing on aspects concerning the methods applied and study design.
Confirmability	Researcher reflexivity was encouraged via the performance of reflexive reports and by describing the rationale behind the study.

## Results

Seventeen grandmothers participated in this study. The mean age was 67.5 years (SD 3.79). The mean number of hours spent per week caring for grandchildren was 24.6 (SD 3.4). In addition, the mean number of days per week spent caring for grandchildren was 5.8 (SD 1.2). See Table 3: Descriptive characteristics of the participants.

**Table 3:** Descriptive characteristics of the participants

Code	Age	Marital status	Occupation	Coexistence	Days of the week care of grandchildren	Hours per week of grandchild care
P1	71	Widow	Housewife	Alone	5	25
P2	67	Married	Housewife	In couple	5	25
P3	63	Married	Housewife	In couple	7	27
P4	67	Married	Housewife	In couple	5	25
P5	64	Married	Retired	In couple	7	28
P6	63	Married	Retired	In couple	7	28
P7	59	Married	Housewife	In couple	5	25
P8	72	Married	Housewife	In couple	7	21
P9	68	Married	Retired	In couple	5	25
P10	69	Married	Retired	In couple	7	28
P11	70	Married	Housewife	In couple	6	24
P12	63	Divorced	Retired	In couple	5	25
P13	67	Widow	Housewife	Alone	5	25
P14	70	Married	Housewife	In couple	5	20
P15	69	Married	Housewife	In couple	6	24
P16	71	Widow	Housewife	Alone	7	28
P17	72	Married	Housewife	In couple	5	25

**Fig. 2:**
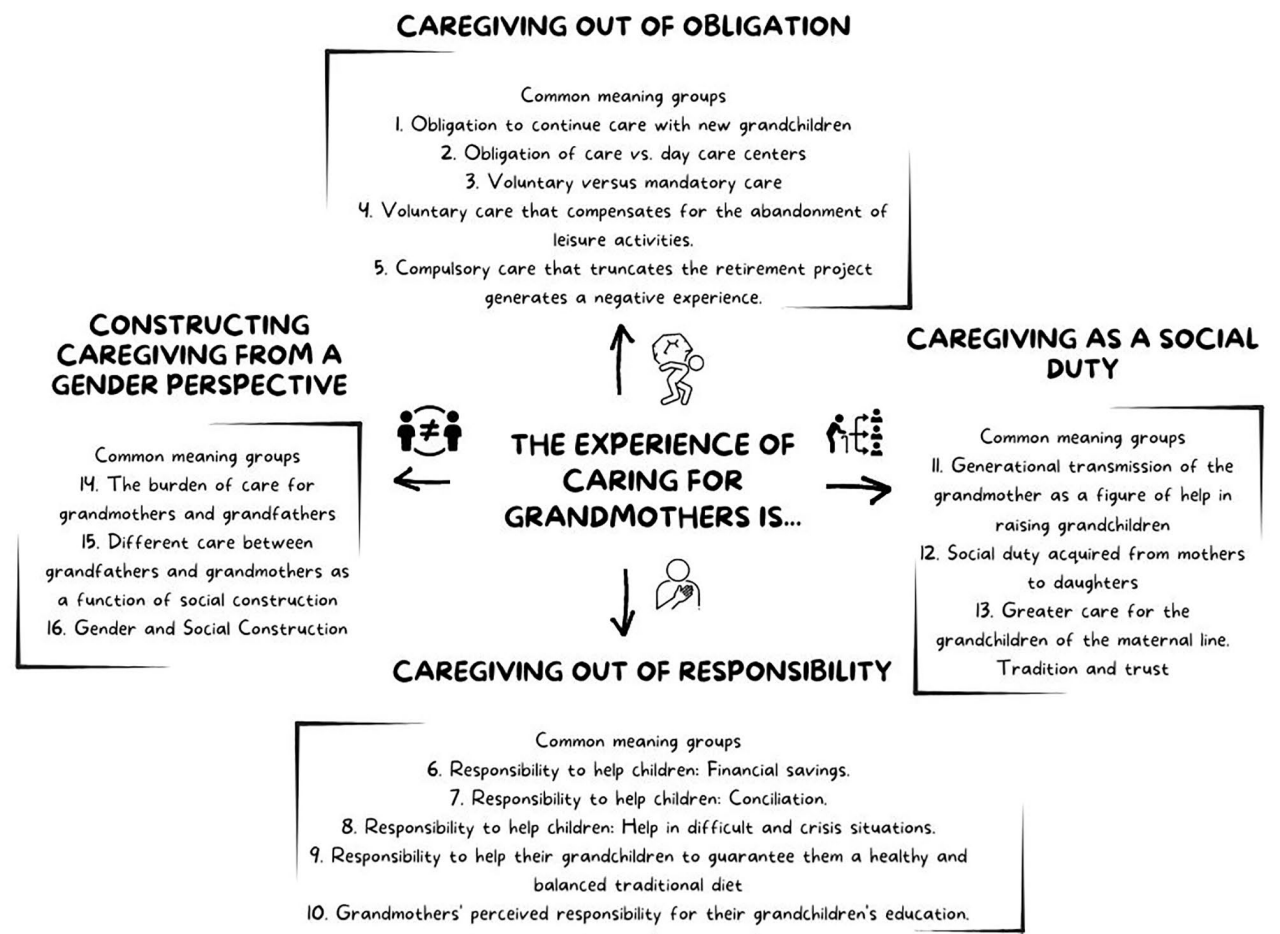
Map of themes and Common meaning groups

Four main themes were identified: (a) Caregiving out of obligation, (b) Caregiving out of responsibility, (c) Caregiving as a social duty, and (d) Constructing caregiving from a gender perspective (Fig. 2). In addition, examples of narratives obtained from participants are included in Appendix 2, presented by themes and meaning clusters.

### Caregiving out of duty and obligation

Participants described feeling a sense of obligation to help their children by caring for their grandchildren. This obligation to provide care applies to all the grandchildren, regardless of the number, understanding that the duty to help all their children is equal. This feeling of duty to help all their children equally, without discrimination, makes them unable to refuse even in the presence of physical limitations.

The grandmothers´ feeling of duty and obligation also appears when it is necessary to take their grandchild to a day care center and/or caregivers outside the family. The reasons for taking care of the grandchildren themselves are that they offer better, more exhaustive and individualized care than a day care center and provide love and affection. Also, avoiding the use of these day care centers saves money. In addition, they do not allow their grandchildren to be taken to a daycare center while they are available.

*“I think I take better care of them. Because in a day care center there are many children*,* I think they don’t take care of them as well as I do. Also*,* I can’t consent to them putting her in daycare while I’m there.” (P7)*.

Duty and obligation are experienced more satisfactorily if the grandmothers voluntarily choose to care for their grandchildren. Voluntary care combined with the feeling of duty to help the family means that it is not perceived as a sacrifice and that the experience is positive. Although grandmothers invest part of “their life” in caring for their grandchildren, understood as the loss of their leisure time and routines, they feel that they have “fulfilled their duty”. On the contrary, it is experienced negatively when the grandmother does not want to do it and it is imposed as “obligatory”.

*“It is a job I have to do*,* it is a feeling*,* not of obligation*,* but that they are part of your life. They are your grandchildren*,* and your daughter is working and what are you going to do*,* we have to make do. And if we have to give up our activities*,* we do. It pays off for me” (P9).*

### Caregiving as a responsibility

The grandmothers described how they have “a responsibility” of generational support. Their help or support have certain purposes such as achieving economic savings for their children by caring for the grandchildren, thus avoiding the hire of external personnel or day care centers. Another purpose is to help their children balance work and family life by helping out with the grandchildren. On occasions, the grandmothers were satisfied to report how their help was instrumental in complementing the family salary and reconciling their working hours and shifts. Still another goal for the grandmothers is that of feeding their grandchildren with which they feel they guarantee a healthy, wholesome and balanced diet for them. And finally, grandmothers aim to educate their grandchildren; the transmission of values is considered a great responsibility.

“*My daughter works and*,* in addition*,* she is studying. Dedication to her work and her child is impossible for her. If I can help my family in any way*,* I must do it and I feel satisfied.”* (P11).

The grandmothers narrated how their role becomes prominent in difficult and crisis situations, in which they do not hesitate to take care of their grandchildren as they feel responsible to help and support the family. Examples of crises are the onset of illness (grandchildren/children), and/or separation/divorce.

“*I started taking care of my granddaughter after a divorce. The child was frightened*,* scared and*,* well*,* how hard it is for children at that time. Your daughter is at home*,* you have to protect her more*,* she is divorced*,* her income has dropped by half. You have to help them*” (P1).

### Caregiving as a social duty

The participants described how they have a social duty, a moral debt acquired when their own mothers took care of their own children. They believe they should reciprocate by helping the following generations. Our participants perceive “their grandmother figure” as a socially accepted and sought-after figure between mothers and daughters. It has always existed, and they contribute to generational continuity through their caregiving. Our participants narrated how their mothers and mothers-in-law were an important help in reconciling their work and personal lives and now want to “pay back” that generational debt.

*“I was working when I had my daughter*,* so my mother took care of her. My mother put her to bed*,* fed her*,* dressed her*,* how can I not do it now for my daughter?” (P3).*

Helping out is not only focused on taking care of the grandchildren, but also includes housework and cleaning. In the majority of the participants, caregiving is mainly provided for the children of the grandmothers ‘daughters, i.e., the maternal lineage. This behavior is a shared norm, which is maintained and perpetuated generationally (from mother to daughter). Greater intimacy and trust between mother and daughter are given as reasons by our participants.

*“ I think that in the case of women there is always a tendency towards her mother. I guess it will be because of the trust. Right now*,* the girl I have belongs to my daughter*,* and I have more confidence to tell her “don’t put those clothes on the girl” and with my daughters-in-law I have kept quiet and have not said anything to them” (P8).*

### Constructing caregiving from a gender perspective

Our participants recounted how the burden of caring for grandchildren falls to them, that is, on the women. Grandmothers take care of their grandchildren, supervise their activities and are ultimately responsible for their upbringing. While grandmothers perform household chores and manage the feeding and hygiene of the grandchildren, the supervisory role may sometimes be relinquished to the grandfather. Grandmothers consider the grandfather to be a “helper” in caring for the grandchildren, but the grandmothers themselves, are ultimately responsible for childcare.

*“He is at home*,* but managing the granddaughter is my job. I take care of everything*,* making the food*,* washing the clothes*,* sewing*,* ironing. Of course*,* he doesn’t do those things*,* because he doesn’t know how. There is a lot of difference between what grandmothers do and what grandfathers do (P9).*

The grandfathers’ participation is centered on leisure-entertainment activities and transportation to school, and their tasks are related to leisure, play and entertainment (going out to the park to play), doing homework. There are also differences in the distribution of tasks by gender in relation to activities outside and inside the home. While grandmothers stay closer to home and take care of activities inside the home, grandfathers are in charge of everything that involves going out of the home with their grandchildren.

*“I do see differences between us and grandfathers They have had that upbringing from our time. They have not been at home like us*,* who are more accustomed to the house and to taking care of things*,* and we manage it better. Because at home*,* I am so comfortable and he is so bored” (P5).*

Our participants recognize that there are gender differences in the distribution of tasks and responsibilities in the care of their grandchildren. They feel they play a mayor and leading role, while grandfathers take on an “auxiliary” role.

## Discussion

Grandmothers feel that they have a duty and obligation to care for their grandchildren in order to help their children. This feeling of duty appears in the work of Badenes & López [33] in which they describe how grandparents feel an obligation to help rather than accept that their grandchildren be cared for by strangers. In situations where their children need outside help to care for their grandchildren, grandmothers feel a deep-rooted sense of responsibility, family commitment and obligation. However, this does not mean that there are no consequences. Previous studies [14, 33, 34] noted that grandparents experience emotional exhaustion when caring for their grandchildren for many hours and they give up other activities or wishes that would be appropriate for an older person. The voluntary aspect of caregiving is described by our participants. Authors such as Broese van Groenou & De Boer [10] and Buchanan & Rotkirch [1], point out that caring for grandchildren is experienced differently depending on whether it derives from a voluntary choice or an obligation. When childcare is not a voluntary choice, previous studies show that the experience can negatively affect the physical and emotional health of grandmothers [10]. Buchanan & Rotkirch [1] observe that caregiving produces satisfaction when it is chosen. But when it is an “imposition” by their children, against their will, or truncates life projects such as retirement, then it becomes a key factor in the presence of feelings of overload. Margolis &Verdery [35] and Lakomý & Kreidl [36] found that grandmothers play a key role in their children´s ability to balance their careers with family life. This help is an attempt by grandmothers to protect the family and support their children in advancing their careers or keeping their jobs. In Spain, Noriega et al. [7] show how grandmothers help many young couples to balance work and family life and also become a significant source of economic support.

Our results coincide with previous studies [14, 37, 38], where grandparents adopt the role of caregivers of their grandchildren, as a tool to help their children prevail over difficulties in reconciling work and family life. The results of the present study also coincide with Bordone et al. [39] who find that this help coming from grandmothers is accentuated in crisis situations such as children’s illnesses, separations, divorces or unemployment. Furthermore, Danielsbacka et al. [40] point out that grandparents perceive themselves as part of a generation whose dedication to the family contributes to the balance and economic sustainability of society. Leeson [41] depicts the role of the grandmother as a fundamental pillar for the maintenance of the family unit. They perform mediation functions in conflicts between their children and grandchildren and serve as a refuge from crisis situations in the family. Lee & Blitz [14] observed that grandmothers not only fulfill the role of helpers in the family. They feel they are “grandmothers for everything”, as they say, because their children resort to the saving figure of grandmothers as the only way to maintain their socioeconomic status and quality of life in the face of any difficulty that may come up.

The helping role to fulfill a debt has been described previously in the work of Di Gessa et al. [9], who identified how grandmothers’ helping role derived from their own observation and past experience with their mothers and their own grandmothers. These authors state that the helping role of grandmothers is progressively constructed as they imitate the role played by their own grandmothers, mothers and mothers-in-law. The involvement of grandmothers in helping families has facilitated the maintenance of their children’s work or professional development. Hill [42] proposes the incorporation of women into the labor market and aging of the population as factors leading grandmothers to take on childcare of their grandchildren. These authors argue that grandparents live longer, have acceptable health and are available to care for their grandchildren. On the other hand, Margolis & Verdery [35] identify the incorporation of both parents into the labor market, inflexible work schedules and the absence of social policies aimed at helping to balance family life and careers as factors that have contributed to the incorporation of grandmothers as an essential resource for families. The presence of generational duty between mothers and daughters is a form of intergenerational family solidarity. For Hank et al. [43] that solidarity consists of providing care and help among family members. Family solidarity is consolidated as fundamental social capital for the well-being of people, in which grandmothers become a key support as they come to the aid of their children and grandchildren in times of need.

Our results detect a gender distribution of tasks between grandmothers and grandfathers in caring for grandchildren. The burden of care and the final responsibility falls to grandmothers, who play a leading role, while the grandfather is an assistant. Our results coincide with Notter [13], who observes that household chores and childcare fall mainly to older women. This means that feminization of old age may perpetuate gender-based caregiving inequities. Differences in the distribution of activities between grandmothers and grandfathers continue to exist today and are based on the social construction that defines his or her roles and participation as caregivers [5]. The participants in the present study point out their importance as protagonists in the care of their grandchildren. Several authors such as Broese van Groenou & De Boer [10] and Notter [13] have observed how greater involvement of grandmothers in caregiving tasks for grandchildren leads to greater satisfaction with the role of caregiver. This factor may explain the grandmothers’ feeling of self-importance, as opposed to the “auxiliary” role assigned to the grandfather. Moreover, Perry & Daly [12] have identified differences between grandmothers and grandfathers as to the performance of caregiving tasks and degree of dedication to and involvement with their grandchildren. These authors have found that grandmothers are the first figure to be asked for help by their children in caring for their grandchildren. In this way, the grandfather becomes a secondary or complementary caregiver.

### Strengths and limitations

One of the strengths of this study was to obtain a profile of our participants, which could be used to explore other dimensions (emotions, feelings, etc.) in other research. This profile consists of women over 65 years of age, with an average age of 68 years, living with their partner, and devoting a large part of their time per week to caring for the grandchildren (25 h and/or 6 days per week on average). The current study has not included the experience of grandfathers. The authors believe it would be of interest to strengthen the present study to include the perspective of grandfathers in order to comprehend the distribution of grandparenting tasks from a gender perspective.

There are different proposals in qualitative research [29]. Specifically, in this study we have focused on studying the lived experience through phenomenology. But it would be an important aspect of future research to study the cultural aspects of care in this group of participants using ethnography, and to analyse in depth the process of caring for grandchildren, which agents are involved (inside and outside the family) in which contexts and circumstances using grounded theory [29, 32].

### Implications for policy and practice

Our results would be of interest to develop support programs for grandmothers aiming to prevent fatigue and exhaustion and support them in caring for their grandchildren. In addition, from policy view it would be necessary to develop studies with a gender perspective on the care provided by grandmothers and grandfathers. It is also essential to promote awareness in various public institutions about the development of intergenerational policies and programmes between grandparents and grandchildren by developing psycho-educational and support programmes where grandparents are helped to: (a) recognise the valuable role of grandparents in today’s society (b) foster social and family cohesion (c) facilitate the transmission of values and strengthen family ties (d) promote active ageing through social participation.

## Conclusions

This research adds knowledge about grandmothers’ experiences of caring for their grandchildren, in terms of their caregiving, motivations, and feelings. Grandmothers are a fundamental pillar of families, enabling the balancing of family and work through their help in caring for grandchildren. Grandmothers embrace the role of caregivers of their grandchildren due to a strong sense of duty, obligation and generational responsibility. However, caregiving tasks were found to be assigned to grandparents according to gender.

These results may help us understand the informal care provided by grandmothers within families and how this caregiving facilitates family life and work for the children’s parents. Knowing and understanding the motivations and experiences of grandmothers in caring for grandchildren will enable the application of comprehensive care. Furthermore, the study of grandmothers’ care of grandchildren and the knowledge of the construction and transmission of care between generations (grandmothers-mothers and daughters) would allow us to understand not only the grandmothers’ experiences, but also to identify the cultural and social factors involved, and how these may influence health and the gender perspective of care.

### Electronic supplementary material

Below is the link to the electronic supplementary material.

Supplementary Material 1

Supplementary Material 2

## Data Availability

The data that support the findings of the study are available on request from the corresponding author, upon reasonable request. The data are not public due to ethics restrictions.
