# Supplementary material for: The role of duty, gender and intergenerational care in grandmothers’ parenting of grandchildren: a phenomenological qualitative study
Source: BMC Nurs. 2024 Jul 15;23:477. doi: 10.1186/s12912-024-02151-0 (PMC11247794; doi:10.1186/s12912-024-02151-0)
Supplement: Supplementary file 1 — Supplementary Material 1 [file 12912_2024_2151_MOESM1_ESM.doc]

**Appendix 1. Interview Guide**.

| Themes | Questions |
| --- | --- |
| Theme 1. Caring by duty and obligation | Sample Question:   - What is it like for you to care for your grandchildren?   Aspects to explore:   - To describe the grandparents' experiences of caring for their grandchildren and their characteristics - Identifying the different reasons for starting to care for grandchildren |
| Theme 2. Caring by responsibility | Sample Question:   - What is your experience of caring for your grandchildren? - What does caring for your grandchildren mean to you in terms of helping your children?   Aspects to explore:   - To describe the different situations that lead grandparents to initiate and maintain care for their grandchildren in relation to the help they give their children in caring for their grandchildren |
| Theme 3. Caring as a social duty | Sample Question:   - How do you see the role of grandmothers?   Aspects to explore:   - Describe the experiences of grandparents in educating and transmitting values to their grandchildren - To describe the social concept of grandmothers for the grandmothers interviewed |
| Theme 4. Building care from a gender perspective | Sample Question:   - What is your experience as a grandmother in caring for your grandchildren? How about as a grandfather?   Aspects to explore:   - To describe the grandmothers' experiences of caring for their grandchildren in relation to the gender differences that exist in grandparents caring for their grandchildren |
