# Supplementary material for: The role of duty, gender and intergenerational care in grandmothers’ parenting of grandchildren: a phenomenological qualitative study
Source: BMC Nurs. 2024 Jul 15;23:477. doi: 10.1186/s12912-024-02151-0 (PMC11247794; doi:10.1186/s12912-024-02151-0)
Supplement: Supplementary file 2 — Supplementary Material 2 [file 12912_2024_2151_MOESM2_ESM.docx]

**Appendix 2**. **Narratives of the participants, divided by themes and groups of meaning**.

| **Theme 1. Caregiving out of duty and obligation** | |
| --- | --- |
| Common meaning groups | Narratives |
| 1. Obligation to continue care with new grandchildren | "If you take care of one grandchild, what do you do with the other daughter's grandchild? When my second daughter got married, and started having children, I told my oldest daughter "I have to take care of your sister's son" You have to give them all a hand, I have to do it, I have to take care of him. I took care of the other grandson, and I have to take care of this one" (P2). |
| 2. Obligation of care vs. day care centers | "Because the parents were working, so instead of her going to daycare, I preferred to take care of her. How could we allow her to take the child to a daycare center at 7 a.m. when we were there" (P4). |
| 3. Voluntary versus mandatory care | "Starting to care for my granddaughter was voluntary. There was no pressure of any kind, simply that she was the only daughter we had. Because I know of people who have grandchildren a little bit out of obligation and they don't take it well. But of course, ours was not an obligation, it was a voluntary thing and it is different" (P6). |
| 4. Voluntary care that compensates for the abandonment of leisure activities. | "I believe that there is time for everything, what is needed is the will to do it. It's a job I have to do, it's a feeling, not of obligation, but that they are part of your life. They are your grandchildren, and your daughter is working and well, what are you going to do, are they going to ask for the bill and come? And what do we grandmothers have to do, we have to make do. And if we have to leave our things, then we do it. It's worth it to me" (P9). |
| 5. Compulsory care that truncates the retirement project generates a negative experience. | "I couldn't have imagined being a grandfather and having to take care of my granddaughter. I thought we were going to have a new life together. My husband and I. In the sense of 'we are free, we have enough to live well, everything is ready for retirement, we have everything prepared'. And suddenly you say "no, no, no, no, it's over and nothing, different life. You are tied up and what do you do, you have to take care of it" (P10). |
| **Theme 2. Caregiving as a responsibility** | |
| Common meaning groups | Narratives |
| 6. Responsibility to help children: Financial savings. | "I believe that grandparents have always been the support for their children and grandchildren, but I believe that now there is a circumstance that has never happened before, we are paying for the crisis . And it is a very big effort, our children now do not even have enough money for day care centers, nor to invest in essential investments such as housing (P12). |
| 7. Responsibility to help children: Conciliation. | "With both parents working, I think it complicates things quite a bit. Either you have someone to lend a hand, or you have to get someone to pick them up for you. In the case of my children that is not possible. They need us" (P9) |
| 8. Responsibility to help children: Help in difficult and crisis situations. | "My daughter is sick with Multiple Sclerosis and it was hard for her to get pregnant. She has to do rehabilitation exercises such as daily swimming and has had a hard time getting and keeping a job, and now she needs me, because the child is very nervous and gives her a lot of work and she needs to rest so she doesn't get flare-ups." (P14) |
| 9. Responsibility to help their grandchildren to guarantee them a healthy and balanced traditional diet. | "And I help them with my food. The food of a lifetime, healthy, Mediterranean food. And that's what the child likes, and I make another little bit for him to take with him for dinner, and many days he takes his dinner and he's so happy, because he loves my food. And it is the healthiest" (P13). |
| 10. Grandmothers' perceived responsibility for their grandchildren's education. | "The role we have in their education is important, I am very clear about that. It is not merely to take care of her, as if you were a person paid to do so. It's your granddaughter and you have a responsibility. And therefore, it is a responsibility that you have to bear" (P15). |
| **Theme 3. Caregiving as a social duty** | |
| Common meaning groups | Narratives |
| 11. Generational transmission of the grandmother as a figure of help in raising grandchildren | "We grandmothers have always helped raise the grandchildren. I have always had my mother's help. Because I also had two, they were 18 months old, but I had my mother at home with me, and she helped me a lot. And now I help my daughter, the same" (P16). |
| 12. Social duty acquired from mothers to daughters | "Mothers always help their children, grandchildren, and whatever is needed. Because if I have been sick, my mother has come and my mother-in-law has even come to help me. And if they have helped us before, now they help us for our children. So, that is the law of life" (P1). |
| 13. Greater care for the grandchildren of the maternal line. Tradition and trust | "For me, they are equal, but you have to recognize that you always pull more for the daughter. Because grandmothers pull more for the daughters than for the sons, that's always the case, you know" (P16). |
| **Theme 4. Constructing caregiving from a gender perspective** | |
| Common meaning groups | Narratives |
| 14. The burden of care for grandmothers and grandfathers | "Now my husband is here, because he has retired and, man, he helps me out at times. Because sometimes he goes to pick her up from school. He always takes her to school at noon. Of course, because he was already retired (P7). |
| 15. Different care between grandfathers and grandmothers as a function of social construction | "Grandpa is there for playtime, you know, grandpa is more in charge of that. Grandmas are more at mealtime, at bath time, or when there is a little problem, or when siblings or cousins get angry with each other, at the limits" (P12). |
| 16. Gender and Social Construction | "So, my husband has not gone to school meetings, nor has he gone to the doctors, nor has he cleaned them or fed them. Like now all the parents go with the children to the doctor, and you see it as normal. My husband, never." (P11) |
